# Optimization of antioxidative peptides from mackerel (*Pneumatophorus japonicus*) viscera

**DOI:** 10.7717/peerj.4373

**Published:** 2018-02-15

**Authors:** Xueqin Wang, Huahua Yu, Ronge Xing, Xiaolin Chen, Song Liu, Pengcheng Li

**Affiliations:** Key Laboratory of Experimental Marine Biology, Institute of Oceanology, Chinese Academy of Sciences, Qingdao, China; Laboratory for Marine Drugs and Bioproducts of Qingdao National Laboratory for Marine Science and Technology, Qingdao, China

**Keywords:** Mackerel viscera, Protease, Response surface methodology, Antioxidant activity

## Abstract

Mackerel (*Pneumatophorus japonicus*) viscera contain large amount of protein. We used five proteases to hydrolyze the viscera, and the hydrolysate treated by neutrase exhibited the highest nitrogen recovery (NR). Then we optimized the preparation conditions for mackerel viscera hydrolysate (MVH) by response surface methodology and investigated the antioxidant activity of MVH. The optimal conditions were as follows: enzyme concentration of 1,762.87 U/g, pH of 6.76, temperature of 43.75 °C, extraction time of 6.0 h and water/material ratio of 20.37 (v/w), and the maximum NR was 37.84%. Furthermore, the molecular weight distribution of MVH was almost below 3,000 Da determined by TSK G2000 SWXL gel filtration chromatography, and the MVH exhibited good antioxidant activities in various *in vitro* assays, including DPPH radical, hydroxyl radical and superoxide anion scavenging activities, reducing power and similar effectivelness as butylated hydroxytoluene and Vitamin E to inhibit lipid peroxidation. The results suggested that MVH could be used as a potential source of antioxidant peptide in food industries.

## Introduction

Marine organisms live in complex habitats and are exposed to extreme conditions, thus producing a wide variety of specific and potent active substances that cannot be found elsewhere. Additionally, the oceans are probably the Earth’s most valuable natural resource, providing food mainly as fish and shellfish (Hamed et al., 2015). In recent years, the demand of fish in all forms is increasing dramatically in the globle market, and over-exploitation of fishery resources has become a major concern worldwide (Choonpicharn et al., 2015; Wilson, Hayes & Carney, 2011). According to the Food and Agricultural Organization (FAO), more than 145.1 million tons of fish are actually caught or farmed annually worldwide (Aissaoui et al., 2017). Nevertheless, fish is a highly perishable product and more than 60% of the by-products generated from fish processing industry are waste, which create burdensome disposal problems and environmental pollution (Cheng, Sun & Wei, 2017). The by-products that include skin, head, tail, viscera and bones are normally used for the production of fishmeal, fishoil, fertilliser, fish silage and animal feed (He, Franco & Zhang, 2013). However, the by-products still contain a significant amount of protein-rich material and can be biotechnological exploited for the production of useful marketable products (Dekkers et al., 2011; Sila & Bougatef, 2016): e.g., Ca-binding peptides from Alaska Pollack (*Theragra chalcogramma*) backbone (Jung et al., 2006), anticoagulant peptides from Striped Seabream (*Lithognathus mormyrus*) viscera (El et al., 2010), antioxidant peptides from Black Pomfret (*Parastromateus niger*) viscera (Ganesh, Nazeer & Kumar, 2011), iron-chelating peptides from Alaska Pollock skin (Guo et al., 2013), anti-hypertensive peptides from Sardine and Tuna heads and viscera (Martinez-Alvarez et al., 2016) and antibacterial hydrolysates from Smooth Hound viscera (Abdelhedi et al., 2016).

Oxidation is a vital process in all living organisms even though it has a side effect of producing free radicals (Di Bernardini et al., 2011). The generated radicals are very unstable and can lead to cell or tissue injuries and various chronic diseases (Cheung, Ng & Wong, 2015). Antioxidants are substances that delay or prevent the oxidation of cellular oxidizable substrates, and many studies focus on finding natural antioxidants from marine fish by-products, since they can protect the human body from free radicals and retard the progress of many chronic diseases. According to Martinez-Alvarez, Chamorro & Brenes (2015), it was shown that fish by-products contained in percentage terms were composed of muscle cuts (15–20%), skin and fins (1–3%), bones (9–15%), heads (9–12%), viscera (12–18%) and scales (5%). Lately, there is a particular interest in researching the antioxidant activity of fish viscera, e.g., Abdelhedi et al. (2017) had obtained low molecular weight (MW) peptide (<1 kDa) by the ultrafiltration from smooth hound viscera protein hydrolysates and the peptide exhibited good antioxidant capacity. Burgos-Hernandez et al. (2016) investigated bioactive fractions from cantabrian anchovy (*Engraulis encrarischolus*) viscera and the fractions showed the antioxidant activity through various assays. In addition, there were many other studies on fish viscera protein hydrolysates (Villamil, Vaquiro & Solanilla, 2017).

Mackerel is one of the most important fishes in China due to its abundance and low cost, and it is rich in high-quality protein resources. In our previous study, we optimized the preparation conditions for mackerel protein hydrolysate (MPH) (Wang et al., 2017), and found the MPH with MW below 2.5 kDa showed the strongest antioxidant activity (Wang et al., 2014b). Furthermore, we purified MPH (<2.5 kDa) and investigated its effect and mechanism for anti-fatigue (Wang et al., 2014a). Although there were many researches on the processing and utilization of mackerel (Morales-Medina et al., 2016; Sampath Kumar, Nazeer & Jaiganesh, 2012; Wang et al., 2013; Wang, Huang & Jiang, 2013), studies were seldom conducted on mackerel by-products such as viscera, head and tail and their biological activities. In order to increase the demand for utilization of mackerel by-products, our study was to optimize the enzymatic protein hydrolysis from mackerel viscera to obtain the antioxidant peptide with the higher nitrogen recovery (NR). Furthermore, the antioxidant activity and molecular-weight distribution of mackerel viscera hydrolysate (MVH) were determined.

## Materials and Methods

### Reagents and chemicals

Five proteases (trypsin, papain, neutrase, acid protease and flavourzyme) were provided by Kangbaotai Co. (Hubei, China). 1,1-Dipheny-2-picryhydrazyl (DPPH), butylated hydroxytoluene (BHT), bovine serum albumin (BSA) and phenazine methosulfate (PMS) were purchased from Sigma Chemical Co. (St Louis, MO, USA). Alpha -tocophero, Nicotinamide-Adenine Dinucleotid (NADH) and Nitroblue Tetrazolium (NBT) were purchased from Ruitaibio Co (Beijing, China). Linoleic acid (≥99%) was purchased from Aladdin Co. (USA). All other chemicals and solvents were of analytical grade.

### Sample collection

Fresh mackerel (*Pneumatophorus japonicus*), 210–260 g/fish, were purchased from a seafood market in Qingdao, China. Whole fish were transported on ice to reduce histamine producing. Upon arrival, the fish were washed and the viscera were collected, minced separately using a grinderand stored at −20 °C until use.

### Proximate composition

The proximate composition was performed according to the AOAC methods of analysis (1984). Moisture was determined by drying the samples in an oven at 105 ± 2 °C for 24 h. The ash content determination was done by incinerating the dried residue of the samples in a muffle furnace overnight at 550 °C. Total lipid content of raw material was quantified by the Soxhlet extraction method with ethyl ether for 7 h. Total protein content was determined by the Kjeldahl analysis (Batista et al., 2010), using nitrogen to protein conversion factor of 6.25.

### Mackerel viscera hydrolysate preparation

Five proteases: trypsin, papain, neutrase, acid protease and flavourzyme, were used in this study to select the optimal one. Viscera were mixed with deionized water at a ratio of 1:10 (w/v). The mixtures were adjusted to the required pH with 0.01mol/L NaOH or HCl and heated in a water bath to the required temperature before proteases were added with the enzyme concentration of 1,000 U/g. The hydrolysis reactions were carried out in a shaking incubator. At the end of the hydrolysis period, the mixtures were heated in boiling water for 10 min to inactivate the proteases. Then the hydrolysates were centrifuged at 18,000 ×*g* (4 °C) for 30 min and the supernatants were stored at 4 °C before use. Among the five hydrolysates, the one with the highest NR was chosen for the further experiment.

### Optimization of MVH preparative conditions

In this section, five major factors (enzyme concentration, pH, extraction temperature, extraction time, water/material ratio) were selected for the single factor experiments. Then, on the basis of the single factor experiments, the five independent variables at five levels were employed in a central composite experimental design (CCD). Design Expert (Trial Version 8.0.6; State-Ease Inc., Minneapolis, MN, USA) was used to analyze and calculate the predicted responses and experimental design for the NR. The responses obtained from each set of experimental designs were analyzed by multiple regressions to fit the following quadratic polynomial model:

\begin{eqnarray*}Y={\beta }_{0}+\sum _{i=1}^{k}{\beta }_{i}{X}_{i}+\sum _{i=1}^{k}{\beta }_{ii}{X}_{i}^{2}+\sum \sum _{i\lt j}{\beta }_{ij}{X}_{i}{X}_{j} \end{eqnarray*}

where *Y* is the response variable, *β*_0_ is a constant, *β*_*i*_, *β*_*ii*_ and *β*_*ij*_ are the linear, quadratic, and interaction coefficients, respectively, while *X*_*i*_ and *X*_*j*_ are the coded independent variables.

According to Design Expert 8.0, the analysis of variance table was generated, and the effect and regression coefficients of linear, quadratic and interaction terms were determined. *P* values greater than 0.05 indicated the model terms were not significant. The regression coefficient was used to perform statistical calculations and the generated 3D surface was from the fitted polynomial equation.

### Determination of the MW distribution

The MW distribution of MVH was determined by gel filtration chromatography on a TSK G2000 SWXL column (7.8 × 300 mm ) with particle size of 5 µm (Toyo Soda, Tokyo, Japan), using high-performance liquid chromatography (HPLC) systerm (Agilent, Santa Clara, CA, USA) with a ultraviolet detector. The mobile phase consisted of 85% (v/v) water, 15% (v/v) acetonitrile (EMD Millipore Co., Billerica, MA, USA). The flow rate was 0.5 ml/min and absorbance was monitored at 214 nm.

The column was calibrated with standard substances (Aladdin Co., Los Angeles, CA, USA): Cytochrome C (12,384 Da), Bovine Pancreas (5,733.49 Da), Thymosin *α*1 (3,108.3 Da), Vitamin B12 (1,355.38 Da), L-Glutathione oxidized (612.638 Da), L-Glutathione (reduced) (307.32 Da) and L-Tyrosine (181.191 Da). The plot of log MW against elution time was constructed and the MW distribution of the MVH was then calculated according to the plot.

### Degree of hydrolysis

The degree of hydrolysis was evaluated as the proportion (%) of a-amino nitrogen with respect to the total nitrogen in the sample (Zhou et al., 2012). Analyses were performed in duplicate.

### Determination of nitrogen recovery

After the hydrolysis reaction, the supernatant was obtained by centrifuging at 4,000×*g* (4 °C) for 20 min. The volume of soluble fraction was recorded and total nitrogen in supernatant was determined using Kjeldahl method. NR was calculated using the following equation (Benjakul & Morrissey, 1997):

\begin{eqnarray*}\text{NR}(\text{%})=\text{total nitrogen in supernatant (mg)/total nitrogen in substrate (mg)}\times 100. \end{eqnarray*}

### Amino acid composition analysis

Amino acid contents weremeasured after acid hydrolysis in accordance with Xu et al. (2015) with some modifications. Hydrolysis of sample was conducted with 6 mol/L HCl at 110 °C for 24 h in a drying oven, and transferred into a 50 ml volumetric flask and diluted to the reticule with distilled water. Then, 1 mL of the filtrate was evaporated at 40–50 °C by rotary evaporator, dissolved in 1–2 mL of distilled water and dried. After complete drying, the residue was diluted with 1 mL of buffer (pH 2.2) and applied to a S433D amino acid analyzer (SYKAM, Eresing, Germany). The amino acids were identified and quantified from standard curves constructed with a mixture standard of threonine (Thr), methionine (Met), valine (Val), isoleucine (Ile), leucine (Leu), phenylalanine (Phe), lysine (Lys), tyrosine (Tyr), histidine (His), arginine (Arg), aspartic acid (Asp), serine (Ser), glutamic acid (Glu), glycine (Gly), alanine (Ala) and proline (Pro) (Sigma Aldrich, St Louis, MO, USA). Because acid hydrolysis oxidises and breaks down tryptophan, these results were not reported.

### Antioxidant activity

#### Hydroxyl radical scavenging activity assay

Scavenging activity of MVH on hydroxyl radicals was performed, using method described by You et al. (2012) with a few modifications. Briefly, the reaction mixture contained 1.0 mL of phosphate buffer (PBS, 0.15 mol/L, pH 7.4), 1.0 mL of safranine T (1.0 mM), 0.5 mL of EDTA-FeSO_4_ (2.0 mmol/L) and 1.0 mL of MVH solution with different concentrations. After sufficient mixing, 1.0 mL of H_2_O_2_ (3%) was added to the mixture. Following incubation at 37 °C for 30 min, the absorbance of the mixture was measured at 520 nm. The hydroxyl radical scavenging activity was calculated as: scavenging rate (%) = [(*A*_1_ − *A*_0_)∕(*A*_2_ − *A*_0_)] × 100, where *A*_1_ was the absorbance of the MVH, *A*_2_ was the absorbance without H_2_O_2_, *A*_0_ was the absorbance of the control. Both *A*_0_ and *A*_2_ were the mixtures with MVH solution replaced by deionized water. All experiments were performed in triplicate.

#### Reducing power assay

The reducing power of the MVH was determined according to the method of Moure, Dominguez & Parajo (2008) with some modifications. A total of 1.0 mL of MVH solution with different concentrations was mixed with 1.0 mL of PBS (0.2 mol/L, pH 6.6) and 1.0 mL 1% (w/v) potassium ferricyanide. The mixture was incubated at 50 °C for 30 min, followed by addition of 2.0 mL 10% (w/v) TCA, 1.25 mL ferric chloride (0.1%, w/v) was added 5 min later and then fully mixed. After 30 min, the absorbance of the mixture was measured at 700 nm. Vitamin C (VC) was used as a positive control. All experiments were carried out in triplicate.

#### DPPH radical scavenging activity assay

The DPPH radical scavenging activities of the MVH were determined as described by Li et al. (2017) with slight modifications. Briefly, 1.0 mL of DPPH (0.1 mmol/L) diluted in ethanol was added to 3.0 mL of MVH solution with different concentrations. After vigorous shaking, the mixture was left to stand for 30 min and the absorbance was measured at 517 nm. The DPPH radical scavenging activity was calculated as follows: scavenging rate (%) = [1 − (*A*_1_ − *A*_0_)∕(*A*_2_ − *A*_0_)] × 100, where *A*_0_ was the absorbance without DPPH, *A*_1_ was the absorbance in the presence of the MVH, and *A*_2_ was the absorbance of the control (without sample). All experiments were performed in triplicate.

#### Superoxide-radical scavenging assay

The superoxide scavenging ability of MVH was assessed based on the method of Li et al. (2012) with a slight modification. The reaction mixture, containing MVH solution with different concentrations, phenazine methosulphate (PMS) (20 mol/L), nicotinamide adenine dinucleotide-reduced (NADH) (240 mol/L) and nitro blue tetrazolium (NBT) (150 mol/L) in Tris–HCl buffer (0.1 mol/L, pH7.4), was incubated at room temperature for 5 min and the absorbance was read at 560 nm against a blank. The capability of scavenging to superoxide radical was calculated using the following equation: scavenging effect (%) = [1 − *A*_1_∕*A*_0_)] × 100, where *A*_1_ was the absorbance in the presence of the sample, and *A*_0_ was the absorbance of the control. VC was used as a positive control. All experiments were carried out in triplicate.

#### Lipid peroxidation in a linoleic acid model system

The method described by Wang et al. (2014b) was used to measure the inhibition of lipid peroxidation in a linoleic acid system with slight modifications. BHT and Vitamin E (VE) were used as positive controls. Briefly, the sample, BHT and VE (5 mg) were dissolved in 10 mL PBS (50 mmol/L, pH 7.0), with 0.13 mL of linoleic acid and 10 mL of 99.5% (v/v) ethanol added in 250 mL conical flasks, the total volume were adjusted to 25 mL with deionized water. The flasks were sealed and incubated at 40 ± 1 °C in dark for eight days. Extent of lipid peroxidation was measured by the ferric thiocyanate method (Wang, Huang & Jiang, 2013; Wang et al., 2013): 0.1 mL of aliquot taken from each flask was mixed with 4.7 mL 75% (v/v) ethanol, 0.1 mL 30% (w/v) ammonium thiocyanate, and 0.1 mL ferrous chloride solution (0.02 mol/L) in 3.5% (v/v) HCl. After 3 min, the absorbance was measured at 500 nm. The blank control group (CK) was the absorbance at 500 nm without MVH. All experiments were carried out in triplicate.

### Statistical analysis

Data were presented as means ± SD. The statistical significance of the data was determined by variance analysis (ANOVA) using the SPSS software (version 18.0 for Windows; SPSS Inc., Chicago, IL, USA) and means were compared by Duncan’s multiple comparison post-test. Statistical differences were considered to be significant at *p* < 0.05.

## Results and Discussion

### Chemical composition

The proximate composition of different part of mackerel was shown in Table 1. The muscle contained higher lipid content and lower ash content, which were 15.31% and 4.19%, respectively; the head and tail contained the highest ash content of 11.21%, comparing to other parts; additionally, the viscera contained higher crude protein and lower lipid content, and could obtain more polypeptides by enzymatic hydrolysis, this is consisted with the result of Ovissipour et al. (2012). Thus, viscera can serve as a good source of protein for various applications.

**Figure 1:**
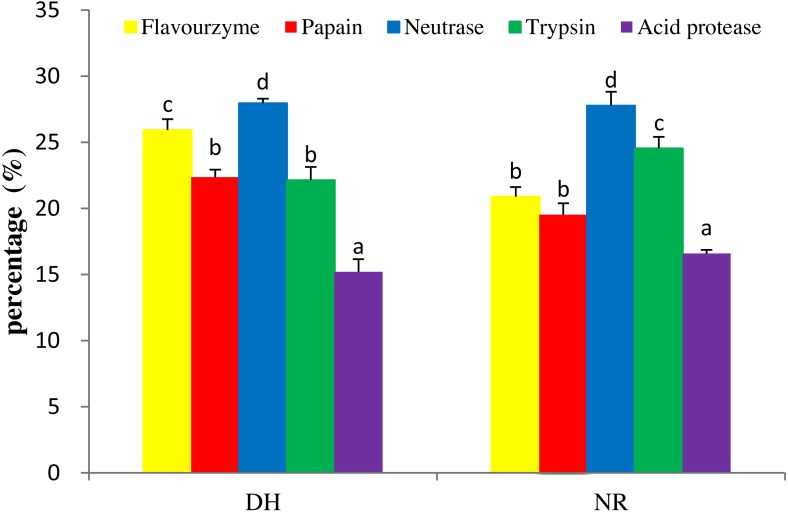
DH and NR of mackerel viscera produced by various proteases. DH, degree of hydrolysis; NR, nitrogen recovery; Data were presented as means ± SD. Different letter indicated significant differences between groups (*P* < 0.05) according to variance analysis (ANOVA) using the SPSS software and means were compared by Duncan’s multiple comparison post-test.

**Table 1:** Chemical composition of mackerel every part.

Each component	%			
	Moisture	Ash	Lipid	Crude protein
Whole fish	72.06 ± 0.91	5.12 ± 0.52	10.05 ± 0.24	16.83 ± 0.24
Muscle	64.55 ± 2.60	4.19 ± 0.59	15.31 ± 0.51	16.66 ± 0.28
Viscera	72.93 ± 0.13	6.41 ± 0.68	5.33 ± 0.16	19.35 ± 0.13
Head and tail	69.78 ± 1.92	11.21 ± 1.07	11.10 ± 0.45	13.24 ± 0.43

**Notes.**

Data were presented as means ± SD.

### Selections of proteolytic enzymes

To produce hydrolysates with desirable properties, it is necessary to undertake studies to find the right proteolytic enzyme for a protein substrate. Five kinds of proteases, i.e., trypsin, papain, neutrase, acid protease and flavourzyme, were used for hydrolysis to produce the target MVH. Generally, the DH and NR were indicators for cleavage of peptide bonds and were used as important parameters that characterized a protein hydrolysate (Wang et al., 2014b). As shown in Fig. 1, the orders of DH and NR for the five hydrolysates as neutrase > flavourzyme > papain > trypsin > acid protease and neutrase > trypsin > flavourzyme > papain > acid protease, respectively. This was consistent with the result of Benjakul & Morrissey (1997), who suggested that the correlation between the DH and NR (*R*^2^ = 0.970–0.978) was high. The result showed that the hydrolysate by neutrase exhibited the highest DH (27.96%) and NR (26.84%) contents compared with other proteases. Therefore, the neutrase-treated hydrolysate was chosen as the best candidate for further studies.

**Figure 2:**
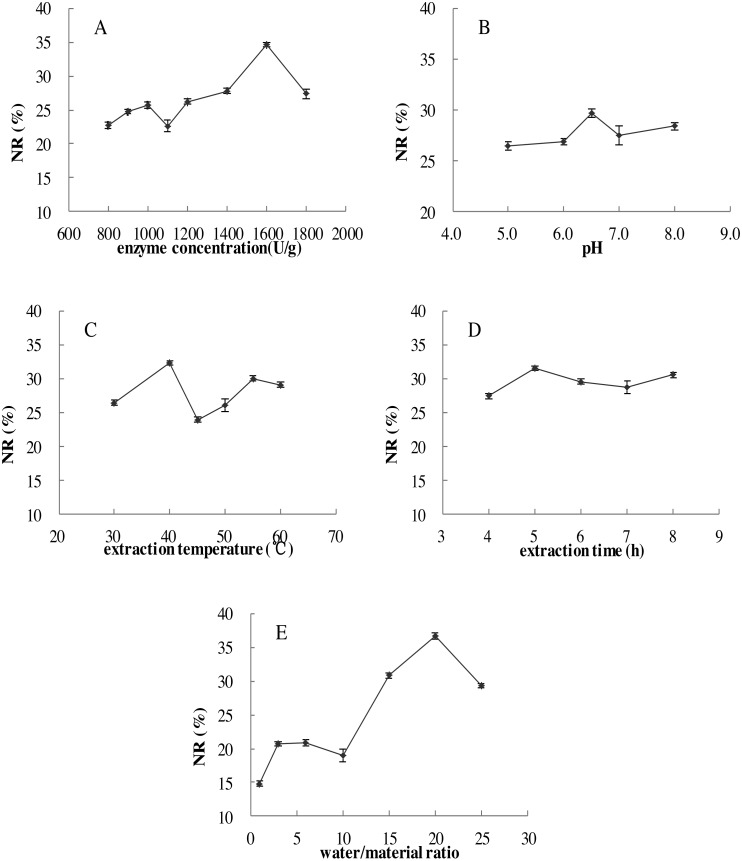
Effects of five single factors on the NR. (A) Enzyme concentration (U/g); (B) pH; (C) extraction temperature (°C); (D) extraction time (h); (E) water/material ratio (v/w) on the NR of MVH; NR, nitrogen recovery. Data were presented as means ± SD.

### Single factor experiments

The effects of five single factors on the NR were investigated in Fig. 2, the result showed that under the range of five single factors, the NR increased to maximum at first and then decreased. The highest levels were found in the range test to obtain NR values and the conditions were displayed: enzyme concentration of 1,600 U/g, pH of 6.5, extraction temperature of 40 °C, extraction time of 5.0 h and water/material ratio of 20 (v/w).

### Optimization of extraction conditions by CCD

According to the single factor experiments, the design matrix and corresponding results obtained from CCD for determining the effects of the five independent variables were listed in Table 2, and the five independent variables (enzyme concentration, pH, extraction temperature, extraction time, water/material ratio) were coded as A, B, C, D and E, respectively.

These results showed that the NR ranged from 29.60% to 37.04%. The data were analyzed via multiple regression analysis using Design-Expert software to yield the following polynomial equation:

\begin{eqnarray*}Y& =& -183.04+0.066\mathrm{A}+30.90\mathrm{B}+1.69C+2.93\mathrm{D}+1.19\mathrm{E}-2.26\times 1{0}^{-4}\mathrm{AE}-0.046\mathrm{CD}\nonumber\\\displaystyle & & +\;0.093\mathrm{DE}-1.76\times 1{0}^{-5}{\mathrm{A}}^{2}-2.30{\mathrm{B}}^{2}-0.016{\mathrm{C}}^{2}-0.22{\mathrm{D}}^{2}-0.034{\mathrm{E}}^{2}. \end{eqnarray*}

Analysis of variance (ANOVA) results for the model were given in Table 3. The corresponding variables were more significant as the *F*-value became greater and the *P* value became smaller (Zhao et al., 2015). Values greater than 0.05 indicated the model terms were not significant.The Model *F*-value of 41.91 implies the model is significant, additionally, it could be seen that the variables with the significant effects (*P* < 0.05) on the NR of MVH were certain linear terms (A, B, C, D and E), quadratic terms (A^2^, B^2^, C^2^, D^2^ and E^2^) and interaction terms (AE, CD and DE). As seen in Table 3, the model showed a good fit with the experimental data, with high values of *R*^2^(95.51%) and Adj. *R*^2^(93.88%). The low coefficient value of the variation (CV = 1.45%) clearly suggested a high degree of precision and reliability of the experimental values. This result implied that the hydrolysis process of MVH could be analyzed and predicted by the model.

The effects of variables and their interactions on NR were illustrated by 3D response surfaces. The figures displayed the effects of two factors on NR while the others were kept at the center point (Feng et al., 2015).

**Figure 3:**
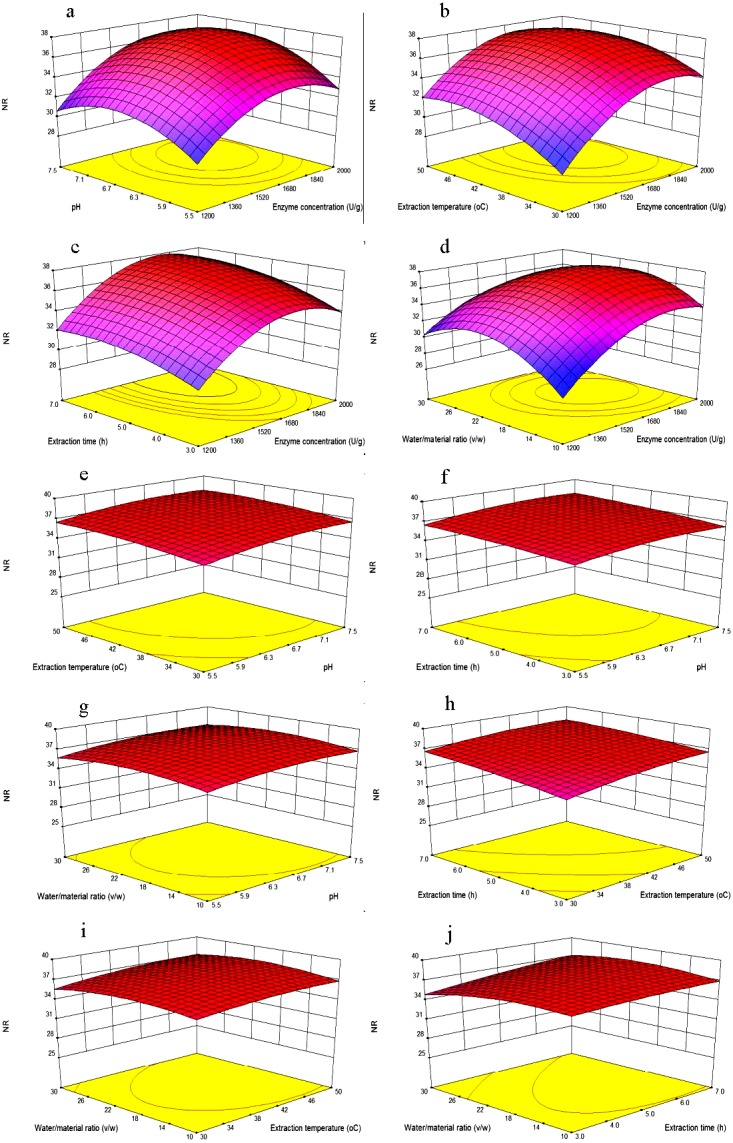
Response surface plots showing the effects of variables. Each figure meant as follows: (A) Effects of enzyme concentration and pH; (B) enzyme concentration and extraction temperature; (C) enzyme concentration and extraction time; (D) enzyme concentration and water/material ratio; (E) pH and extraction temperature; (F) pH and extraction time; (G) pH and water/material ratio; (H) extraction time and extraction temperature; (I) extraction temperature and water/material ratio; (J) extraction time and water/material ratio on the NR of MVH; NR, nitrogen recovery.

Figure 3A showed that NR increased as the enzyme concentration was increased from 1,200 to 1,800 U/g, that because in higher enzyme concentration, there would be more chances for the hydrolysis to occur. However, the NR was no longer increased as the enzyme concentration was 2,000 U/g. When the mackerel viscera were consumed by the neutrase, the excess enzyme might not participate in the reaction, once a certain level of enzyme was reached, there would be a plateau on the NR levels. When the pH increased from 5.5 to 7.5, NR increased firstly and then decreased. Because each protein has different isoelectric point, and different pH value might affect the solubility of protein.

As shown in Fig. 3B, at lower enzyme concentrations, when temperature increased from 30 °C to 45 °C, the NR increased because of increasing of temperature would help to the spread of solute and increase of yield. However, the NR would no longer increase when the extraction temperature increased to 50 °C. This was likely because high extraction temperatures may lead to denaturation and inactivation of enzymes (Feng et al., 2015). When enzyme concentrations increased from 1,200 to 1,800 U/g, NR increased significantly, while too high enzyme concentration no longer increased the NR. Maximum NR was achieved when the extraction temperature and enzyme concentrations were 45 °C and 1,800 U/g, respectively.

**Table 2:** Experimental design and result of response surface.

Run numbers	A Enzyme concentration (U/g)	B pH	C Extraction temperature (°C)	D Extraction time (h)	E Water/material ratio (v/w)	NR (%)
1	1,400	6	35	4	15	30.97
2	1,800	6	35	4	15	33.55
3	1,400	7	35	4	15	31.58
4	1,800	7	35	4	15	34.31
5	1,400	6	45	4	15	33.85
6	1,800	6	45	4	15	34.92
7	1,400	7	45	4	15	34.16
8	1,800	7	45	4	15	35.83
9	1,400	6	35	6	15	31.27
10	1,800	6	35	6	15	34.61
11	1,400	7	35	6	15	32.64
12	1,800	7	35	6	15	35.98
13	1,400	6	45	6	15	33.25
14	1,800	6	45	6	15	35.07
15	1,400	7	45	6	15	33.70
16	1,800	7	45	6	15	37.04
17	1,400	6	35	4	25	29.60
18	1,800	6	35	4	25	31.12
19	1,400	7	35	4	25	30.11
20	1,800	7	35	4	25	32.38
21	1,400	6	45	4	25	32.13
22	1,800	6	45	4	25	33.90
23	1,400	7	45	4	25	33.14
24	1,800	7	45	4	25	34.92
25	1,400	6	35	6	25	32.13
26	1,800	6	35	6	25	33.65
27	1,400	7	35	6	25	33.90
28	1,800	7	35	6	25	34.92
29	1,400	6	45	6	25	34.66
30	1,800	6	45	6	25	35.67
31	1,400	7	45	6	25	34.92
32	1,800	7	45	6	25	36.69
33	1,200	6.5	40	5	20	30.36
34	2,000	6.5	40	5	20	37.04
35	1,600	5.5	40	5	20	33.40
36	1,600	7.5	40	5	20	35.02
37	1,600	6.5	30	5	20	33.19
38	1,600	6.5	50	5	20	36.64
39	1,600	6.5	40	3	20	34.21
40	1,600	6.5	40	7	20	37.04
41	1,600	6.5	40	5	10	33.80
42	1,600	6.5	40	5	30	32.49
43	1,600	6.5	40	5	20	35.83
44	1,600	6.5	40	5	20	36.43
45	1,600	6.5	40	5	20	36.43
46	1,600	6.5	40	5	20	36.23
47	1,600	6.5	40	5	20	36.84
48	1,600	6.5	40	5	20	36.84
49	1,600	6.5	40	5	20	36.23
50	1,600	6.5	40	5	20	36.23

**Notes.**

NR meant nitrogen recovery, and data represented mean of three measured values.

**Table 3:** ANOVA for response surface quadratic model.

Variables	Sum of squares	DF	Mean square	*F* value	*P* value^*^
Model	188.53	13	14.50	58.87	<0.0001
A	52.66	1	52.66	213.76	<0.0001
B	9.10	1	9.10	36.93	<0.0001
C	36.10	1	36.10	146.55	<0.0001
D	21.46	1	21.46	87.11	<0.0001
E	3.30	1	3.30	13.39	0.0008
AE	1.64	1	1.64	6.64	0.0142
CD	1.68	1	1.68	6.83	0.0130
DE	6.87	1	6.87	27.88	<0.0001
A^2^	15.79	1	15.79	64.09	<0.0001
B^2^	10.61	1	10.61	43.08	<0.0001
C^2^	5.09	1	5.09	20.66	<0.0001
D^2^	1.57	1	1.57	6.38	0.0160
E^2^	22.66	1	22.66	92.00	<0.0001
*R*^2^	0.9551				
Adj. *R*^2^	0.9388				
CV%	1.45				

**Notes.**

* Values greater than 0.05 indicated the model terms were not significant.

As shown in Fig. 3C, NR increased when the extraction time increased from 3 to 6 h, and then become flat with the extraction time of 7 h, we inferred that the hydrolysis reaction was powerful in the first four hours, and become flat later. Then, NR would no longer increase with the extraction time when diffusion equilibrium reached. In Fig. 3D, NR increased significantly with the water/material ratio increasing from 10 to 22 (v/w), and decreased significantly when the water/material ratio was 30 (v/w). This result was consistent with the reported from Feng et al. (2015). We speculated that higher water/material ratio may dilute the enzyme concentration, and slow down the rate of enzyme reaction.

Figures 3E, 3F and 3H showed that with the increasing of any two factors between pH and extraction temperature and extraction time, NR was increased slowly with the value from about 34.5% to 37.5%. Additionally, as shown in Figs. 3G, 3I and 3J, when the pH and extraction temperature and extraction time increased, respectively, NR decreased slowly with the water/material ratio increasing from 10 to 30 (v/w), because the higher water/material ratio led to a faster decrease in the number of active catalyst molecules. The variation trends of water/material ratio were different between Figs. 3G and 3D. Because the different other independent variables were kept at zero levels, the different two continuous variables were obtained.

Using Design-Expert 8.0, the optimal hydrolysate conditions of MVH were enzyme concentration of 1,762.87 U/g, pH of 6.76, temperature of 43.75 °C, extraction time of 6.0 h and water/material ratio of 20.37 (v/w). The maximum NR was 37.84%, which was in agreement with the experimental value (37.50%) within a 95% confidence interval, suggesting a good fit between the model and experimental data.

### Amino acid composition

The amino acid composition of MVH is shown in Table 4, and the MVH was rich in Glu, Lys, Leu and Arg. Suetsuna, Ukeda & Ochi (2000) reported that Glu, Lys, Tyr, and Leu contribute to the potency of antioxidant peptides, and MVH was presumed to have antioxidant activity. Additionally, the ratios of essential amino acids to non-essential amino acids and essential amino acids to total amino acids were 0.72 and 0.42, respectively; both ratios are above the FAO/WHO recommended values. The result showed that the components of MVH were balanced, and it would be potential source of bioactive peptides.

### Molecular-weight distribution of MVH

**Figure 4:**
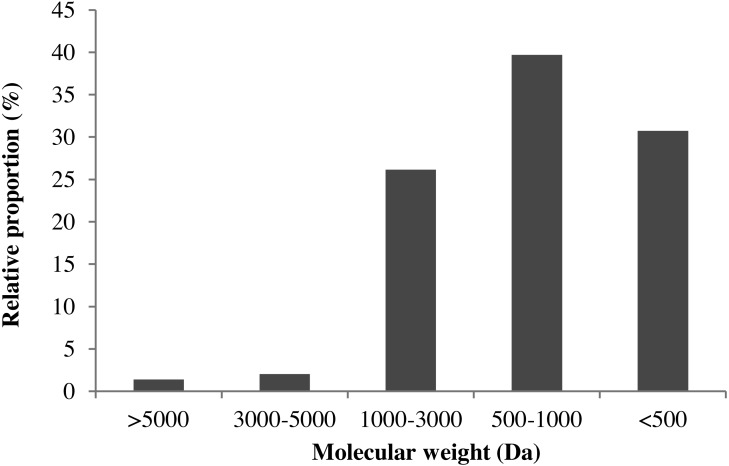
The molecular-weight distribution of MVH.

Many studies have confirmed that the bioactive peptides below 3000 Da exhibited higher antioxidant activity (Ahn, Je & Cho, 2012; Dong et al., 2010). In this section, TSK G2000 SWXL column (7.8 × 300 mm) was used to study the molecular-weight distribution profiles of MVH. As shown in Fig. 4, MVH was divided into thirteen components, and the area of each peak was quite different.

**Table 4:** Amino acid compositions of MVH.

Amino acid	MVH (%)
Asp	3.19
Thr^a^	1.63
Ser	1.99
Glu	5.70
Gly	2.75
Ala	3.03
Val^a^	2.54
Met^a^	1.25
Ile^a^	3.10
Leu^a^	3.50
Tyr	1.33
Phe^a^	2.30
Lys^a^	4.25
His	0.44
Arg	6.05
Pro	1.18
∑AA	44.23
∑EAA	18.57
∑EAA/∑NEAA	0.72
∑EAA/∑AA	0.42

**Notes.**

a Expressed Essential amino acids.

∑AA expressed total amino acids content ∑EAA expressed essential amino acids content ∑NEAA expressed non-essential amino acids content

The retention time (*x*) as abscissa, the corresponding logarithm of molecular weight (*y*) as ordinate, a linear regression equation derived for the standard curve: *y* =  − 0.3602*x* + 13.8, *R*^2^ = 0.9957. According to the regression equation, the molecular-weight distribution of MVH was given. The relative proportion of MW >3,000 Da was only 3.41%, and MW of MVH was almost lower than 3000 Da through hydrolytic reaction. This result was consistent with that reported by Dong et al. (2008). Additionally, Samaranayaka & Li-Chan (2011) had suggested that the range of 500–3,000 Da was a crucial factor affecting the antioxidant activity of protein hydrolysates.

### Antioxidant activity of MVH

There are many evaluation assays of antioxidant activity (Liu et al., 2017). In this section, MVH was examined for ability to protect against oxidation by four *in vitro* assays, including DPPH radical, hydroxyl radical and superoxide anion scavenging activities, reducing power and linoleic acid peroxidation system. As shown in Table 5, the IC_50_ value of MVH for DPPH radicals was 0.81 mg/mL, the result was higher than those reported by Teixeira et al. (2016), which showed that the IC_50_ value of Cape hake by-products hydrolysated (HPH) for DPPH radicals was 4.2 mg/mL, additionally, IC_50_ values of MVH for hydroxyl radicals and superoxide anion were 0.52 and 0.46 mg/mL, respectively, which was also higher than other studies (Han et al., 2015; Teixeira et al., 2016). Besides, the IC_50_ values of VC for DPPH radicals, hydroxyl radicals and superoxide anion were 2.6-fold, 5.87-fold and 3.17-fold of MVH, respectively. The result showed MVH generally demonstrated an excellent ability to scavenge free radicals. Additionally, the concentration of MVH was 1.35 mg/mL when the A_700_ was 0.5, which indicated the MVH had a strong ability to reduce ferric ions to ferrous ions.

**Table 5:** *In vitro* antioxidant activities of MVH.

Sample	IC_50_ value (mg/mL)			
	Hydroxyl	Superoxide	DPPH	Reducing power^a^ (mg/mL)
MVH	0.52 ± 0.02a	0.46 ± 0.11a	0.81 ± 0.08a	1.35 ± 0.01a
VC	3.05 ± 0.06b	1.46 ± 0.23b	2.11 ± 0.05b	1.98 ± 0.06b

**Notes.**

Data were presented as means ± SD. Values followed by a different letter in the same columns were significantly different (*P* < 0.05) ccording to variance analysis (ANOVA) using the SPSS software and means were compared by Duncan’s multiple comparison post-test.

a The concentration of sample at the A_700_ was 0.5.

**Figure 5:**
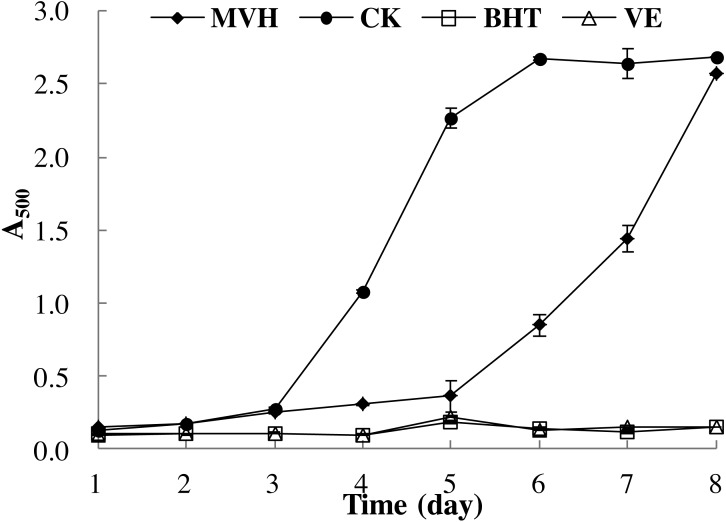
Lipid peroxidation inhibition activity of MVH. mackerel viscera. Data were presented as means ± SD.

The linoleic acid peroxidation system could reflect the multiple mechanisms by which samples may act as antioxidants to retard or inhibit lipid oxidation in a food system (Cheung et al., 2012). Yun et al. (2016) had indicated that the oxidative damage mainly due to the lipid peroxidation reaction between polyunsaturated fatty acid (PUFA) and the free radicals. Therefore, in this section, the ability of MVH to suppress lipid peroxidation in a linoleic acid model system was investigated. Low A_500_ value meat the sample had high lipid peroxidation inhibition ability. As shown in Fig. 5, it is clear that MVH demonstrated an ability to delay linoleic acid peroxidation. This inhibition of MVH was consistently maintained up to five days compared to the control group without an antioxidant, and close to the BHT and VE groups. Han et al. (2015) had investigated the lipid peroxidation inhibition ability of yellowfin tuna (*Thunnus albacares*) skin gelatin hydrolysate (GHs) prepared by neutrase, and the A_500_ value was about 0.50 in the first three days. While the A_500_ value of MVH was nearly 0.25 in the first three days, which was singificant lower than GHs. From the sixth day, the lipid peroxidation inhibition ability of MVH was slightly decreased, and was continued to decrease until the experiment ended. Even though MVH displayed the weakest protection against oxidation of linoleic acid, in comparison to the control group, it still delayed the onset of lipid oxidation from the sixth to the eighth day. Overall, MVH showed good DPPH radical, hydroxyl radical and superoxide anion scavenging activities, ferric ion reducing power and excellent lipid peroxidation inhibition ability.

## Conclusion

In this work, conditions of peptide extraction from mackerel viscera was optimized, and the optimum extraction conditions were as follows: enzyme concentration of 1,762.87 U/g, pH of 6.76, temperature of 43.75 °C, extraction time of 6.0 h and water/material ratio of 20.37 (v/w), and the maximum NR was 37.84%. The most appropriate concentration of enzymes was below 1,800 U/g, and higher enzyme concentration would no longer increase the NR level. Furthermore, temperatures above 45 °C may lead to denaturation and inactivation of enzymes, and no longer increase the NR. Additonally, pH more than 7.0 and water/material ratio exceed 25 (v/w) could significantly reduce the NR. Molecular-weight distribution of MVH was almost below 3,000 Da, and the antioxidant activity by five *in vitro* assays of MVH was determined. Comparing the positive control, MVH exhibited higher antioxidant activity and could be used as a natural antioxidant in enhancing antioxidant properties of functional foods.

##  Supplemental Information

10.7717/peerj.4373/supp-1 Supplemental Information 1 Raw data of the Figures and Tables

## Additional Information and Declarations

### Data Availability

The following information was supplied regarding data availability: The raw data is provided as a Supplemental File.
